# Rapid diagnosis of bacterial vaginosis using machine-learning-assisted surface-enhanced Raman spectroscopy of human vaginal fluids

**DOI:** 10.1128/msystems.01058-24

**Published:** 2024-12-10

**Authors:** Xin-Ru Wen, Jia-Wei Tang, Jie Chen, Hui-Min Chen, Muhammad Usman, Quan Yuan, Yu-Rong Tang, Yu-Dong Zhang, Hui-Jin Chen, Liang Wang

**Affiliations:** 1 School of Medical Informatics and Engineering, Xuzhou Medical University 38044 , Xuzhou, Jiangsu, China; 2 Department of Laboratory Medicine, Shengli Oilfield Central Hospital 499782 , Dongying, Shandong, China; 3 Department of Laboratory Medicine, Guangdong Provincial People’s Hospital (Guangdong Academy of Medical Sciences), Southern Medical University, Guangzhou, China; 4 Dongying’s Leading Laboratory for Hematology, Dongying, Shandong, China; 5 School of 1st Clinical Medicine, Xuzhou Medical University 38044 , Xuzhou, Jiangsu, China; 6 Division of Microbiology and Immunology, School of Biomedical Sciences, The University of Western Australia, Crawley, Western Australia, Australia; 7 Centre for Precision Health, School of Medical and Health Sciences, Edith Cowan University, Western Australia, Australia; Katholieke Universiteit Leuven, Leuven, Belgium

**Keywords:** SERS, machine learning, bacterial vaginosis, deep learning, rapid identification

## Abstract

Bacterial vaginosis (BV) is an abnormal gynecological condition caused by the overgrowth of specific bacteria in the vagina. This study aims to develop a novel method for BV detection by integrating surface-enhanced Raman scattering (SERS) with machine learning (ML) algorithms. Vaginal fluid samples were classified as BV positive or BV negative using the BVBlue Test and clinical microscopy, followed by SERS spectral acquisition to construct the data set. Preliminary SERS spectral analysis revealed notable disparities in characteristic peak features. Multiple ML models were constructed and optimized, with the convolutional neural network (CNN) model achieving the highest prediction accuracy at 99%. Gradient-weighted class activation mapping (Grad-CAM) was used to highlight important regions in the images for prediction. Moreover, the CNN model was blindly tested on SERS spectra of vaginal fluid samples collected from 40 participants with unknown BV infection status, achieving a prediction accuracy of 90.75% compared with the results of the BVBlue Test combined with clinical microscopy. This novel technique is simple, cheap, and rapid in accurately diagnosing bacterial vaginosis, potentially complementing current diagnostic methods in clinical laboratories.

**IMPORTANCE:**

The accurate and rapid diagnosis of bacterial vaginosis (BV) is crucial due to its high prevalence and association with serious health complications, including increased risk of sexually transmitted infections and adverse pregnancy outcomes. Although widely used, traditional diagnostic methods have significant limitations in subjectivity, complexity, and cost. The development of a novel diagnostic approach that integrates SERS with ML offers a promising solution. The CNN model’s high prediction accuracy, cost-effectiveness, and extraordinary rapidity underscore its significant potential to enhance the diagnosis of BV in clinical settings. This method not only addresses the limitations of current diagnostic tools but also provides a more accessible and reliable option for healthcare providers, ultimately enhancing patient care and health outcomes.

## INTRODUCTION

Bacterial vaginosis (BV) represents one of the most prevalent abnormal conditions of the female vaginal environment, posing significant risks for women in their reproductive years, as well as for their offspring and partners (1). In particular, BV has negative impacts on reproductive health outcomes, such as pelvic inflammatory disease, miscarriage, and preterm birth, and facilitates an increased risk of HIV infection and subsequent transmission (2). Therefore, accurate diagnosis of BV is of great significance for its prevention and early intervention. Currently, the gold standards for BV diagnosis include the Amsel criteria and the Nugent scoring methods, which have been routinely used in clinical laboratories for decades. The Amsel criteria, widely utilized in clinical settings, are based on the observation of abnormalities in vaginal discharge, an elevated pH (>4.5), a characteristic odor triggered by the addition of potassium hydroxide, or the detection of clue cells through Gram staining (3). As for the Nugent scoring method, it requires a meticulous enumeration and scoring of various bacterial types and their quantities present in vaginal smears (4). These methods are time-consuming and labor-intensive, the results of which rely on the skills and experience of laboratory staff. Recently, many studies have confirmed the potential of the BVBlue Test in detecting and diagnosing bacterial vaginosis (5–7). Clinically, the technique is highly valued due to its rapidness, simplicity, and specificity, enabling prompt field diagnosis through a specific relative amplification (8). Specifically, it functions by inducing a chemical reaction between vaginal fluid samples and reagents within a tube. In the presence of bacterial vaginosis, the reagent’s color changes to blue or green. In the 2018 Guidelines for the Management of Vaginal Discharge (Vaginal Infections) by the European International Union against Sexually Transmitted Infections and the World Health Organization, the use of commercial BVBlue kits is also recommended as a supplementary diagnostic tool (9). However, in actual operation, sometimes the color of the sample turns gray, making it challenging to determine the diagnostic result. Therefore, the development of novel diagnostic methods with rapidity and accuracy for bacterial vaginosis remains needed.

As an emerging technique, surface-enhanced Raman spectroscopy (SERS) shows highly sensitive and high-resolution capacity during the analysis of clinical samples (10). By leveraging the principle of surface plasmon resonance to enhance Raman scattering, the technique strengthens the interactions between incident light and clinical samples, significantly amplifying the Raman scattering signals and yielding detailed information about the chemical compositions and molecular vibrations of clinical samples (11). This has greatly improved the accuracy of disease diagnosis and does not need complex sample preprocessing procedures by highly skilled laboratory staff (12). The application of SERS technology in the analysis of body fluid samples such as saliva, blood, urine, and vaginal secretions suggests that the technique has potential in the early detection and diagnosis of human diseases (13). However, there are currently few studies on vaginal secretions, and further exploration and development are still needed.

Due to the high complexity and similarity of SERS spectra generated from clinical samples, classical statistical analysis is not sufficient for data analysis, and machine learning (ML) algorithms are regularly used for spectral classification and prediction (14–16). Various ML algorithms have been introduced, including, but not limited to, support vector machine (SVM), decision tree (DT), and convolutional neural network (CNN), to improve the accuracy of identification through SERS spectroscopy (17–19). Although ML algorithms have demonstrated significant advantages in the analysis of Raman spectra, the mechanisms behind the models’ high-accuracy predictions often lack transparency, restricting the understanding of their performance (20). The gradient-weighted class activation mapping (Grad-CAM) algorithm proposed by Selvaraju et al. confers “interpretability” to numerous CNN-based classification models (21). Therefore, the algorithm was also applied to interpret the mechanisms of the CNN model prediction process in this study.

A total of 100 female participants were recruited, and their vaginal fluids were collected and divided into BV-negative (*N* = 50) and BV-positive (*N* = 50) groups based on the results of the BVBlue Test and the clinical microscopy. The vaginal fluids were then used to generate SERS spectra for further spectral and computational analysis. Seven ML models were constructed based on these SERS spectra, including CNN, SVM, random forest (RF), extreme gradient boost (XGBoost), gradient boost (GBoost), AdaBoost, and DT. Our results showed that all the models achieved a prediction accuracy of over 90%. Among these models, the CNN model demonstrated the best predictive capacity, achieving 99% accuracy and an area under the curve (AUC) close to 1 in distinguishing BV-positive from BV-negative samples. To interpret the predictability of the model, we further employed the Grad-CAM algorithm to visualize the impact of each layer of the CNN model during the prediction process. Subsequently, we collected extra vaginal fluid samples (*N* = 40) and applied the optimally trained CNN model for BV prediction, the results of which were compared with the test results using BVBlue Test and clinical microscopy. The blind test results showed an overall prediction accuracy of 90.75%, with a prediction accuracy of 94.25% for positive samples and 87.25% for negative samples. Taken together, this study confirmed the effectiveness of the SERS technique combined with ML algorithms in the rapid and accurate prediction and discrimination of BV-negative and BV-positive samples, demonstrating its potential as a novel diagnostic method for bacterial vaginosis in clinical laboratories.

## RESULTS

### Characterization of silver nanoparticles

**Fig 1:**
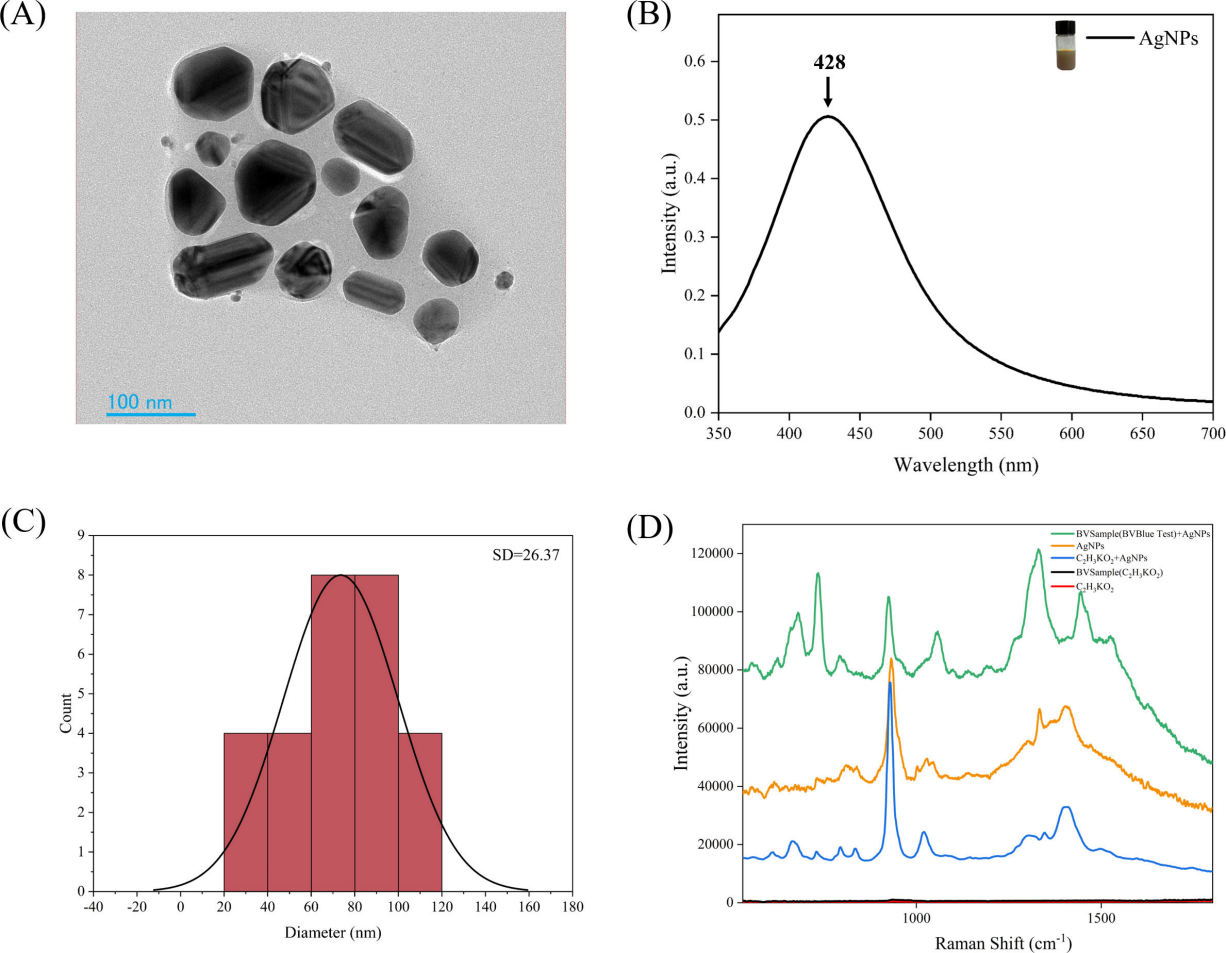
Structure and properties of silver nanoparticles. (**A**) TEM image of AgNPs. (**B**) The absorption spectrum of AgNPs; the characteristic absorption peak is located at 428 nm. (**C**) Histogram of the diameter distribution of silver nanoparticles. The red bars in the figure represent the number distribution of particles with different diameters, and the covered black curve is the Gaussian fit of the distribution, with an SD of 26.37 nm, used to describe the width of the particle size distribution. (**D**) SERS spectral curves of different solutions. Each curve represents the Raman scattering intensity as a function of Raman shift for a specific compound.

At the beginning of our study, silver nanoparticles (AgNPs) were prepared and characterized using transmission electron microscopy (TEM) and ultraviolet-visible (UV-Vis) spectroscopy (Fig. 1A and B). The TEM images revealed the morphology of the silver nanoparticles, and the characteristic peaks in the UV-Vis spectra further confirmed the presence of AgNPs. The symmetry and sharp shape of these peaks indicate a certain degree of uniformity and monodispersity of the particles. This observation is consistent with the findings of Hou et al. (22). Subsequently, the particle size distribution of the silver nanoparticles was analyzed using software ImageJ (version 1.53q). The results showed that most particle sizes ranged between 60 and 120 nm, with a particular concentration around 80 nm (Fig. 1C). We compared the signals between BV samples, silver nitrate, and potassium acetate buffer to determine the effectiveness of silver nitrate in enhancing the SERS signal. The SERS spectra (Fig. 1D) demonstrated that the silver nanoparticles induced strong molecular vibrations in BV samples, confirming that AgNPs can significantly improve the Raman spectral signal of samples. Therefore, in subsequent studies, AgNPs can be considered an effective substrate for SERS analysis.

### Average SERS spectra and deconvolution analysis

**Fig 2:**
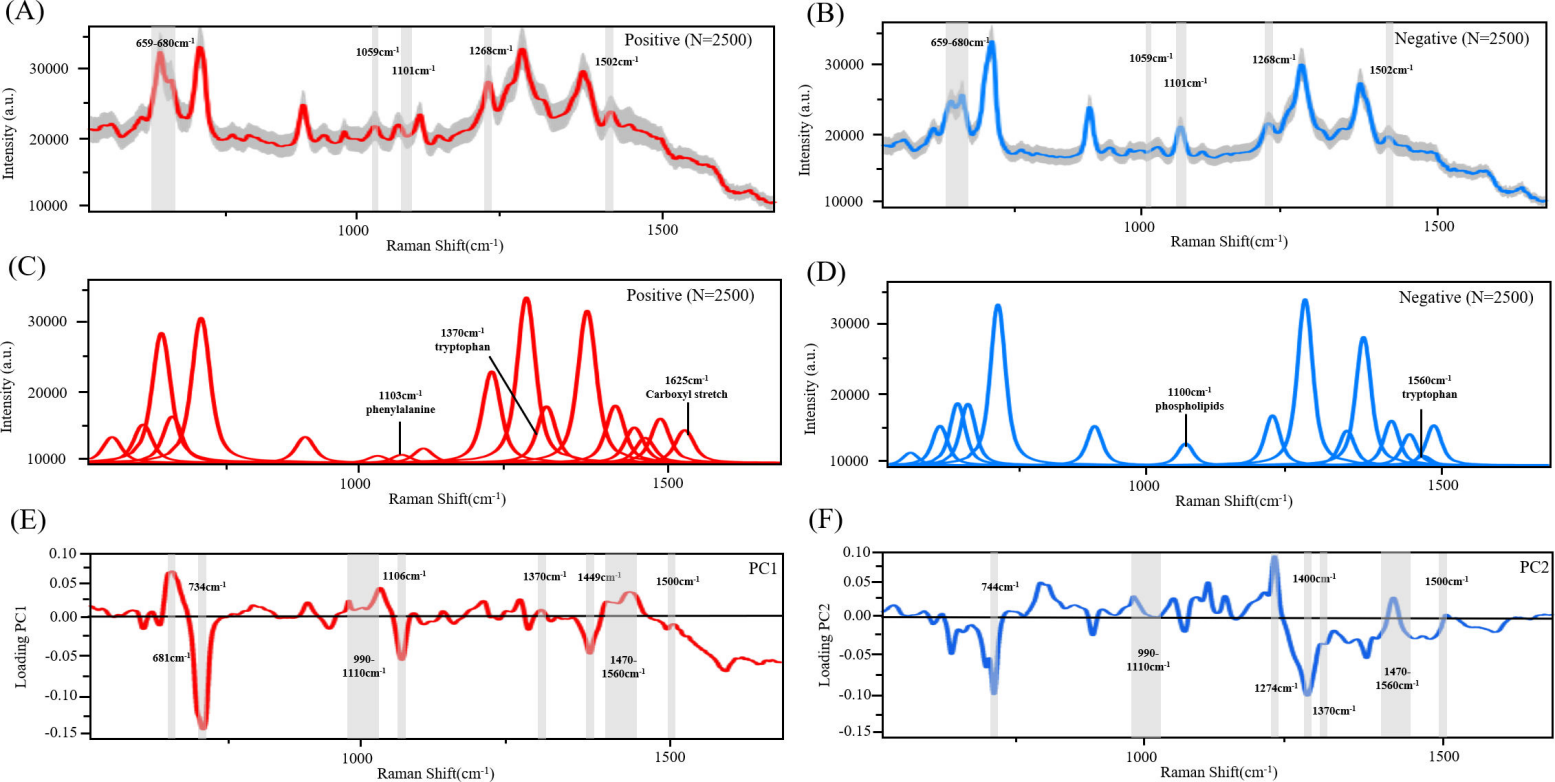
SERS and PCA analysis. (**A** and **B**) SERS fingerprints of BV-positive and BV-negative samples. (**C** and **D**) Deconvolution spectra of positive and negative SERS signals. (**E** and **F**) Loading plots for positive and negative SERS signals.

SERS spectra can reflect subtle differences between clinical body fluid samples and can display structural information with SERS signal intensities at different Raman shifts (23). A total of 5,000 SERS spectra were obtained from collected BV-positive (*n* = 50) and BV-negative (*n* = 50) samples, with 2,500 spectra in each group. Figure 2A and B shows the average SERS spectra of positive and negative samples, where the gray area represents 20% standard deviation (SD) to reveal the variability or dispersion in the data (24). The results show differences between the average spectra of BV-positive and BV-negative samples. In the average spectrum, the characteristic peak at 659–680 cm^−1^ shows stronger vibrations in positive samples compared with negative samples. Advanced deconvolution mapping techniques can be used to conduct an in-depth and comprehensive analysis of raw spectral data, thereby uncovering complex patterns and features that were previously hidden (25). Each sub-band represents a spectral characteristic peak (Fig. 2C and D). Distinct characteristic peaks can be observed between the two groups of samples, and the metabolic diversity is greater in the BV-positive samples. We generated 2D and 3D principal component analysis (PCA) scatter plots (Fig. S1) to assess the separation between the different classes. However, the lack of clear separation in both the 2D and the 3D visualizations suggests that the variance captured by the first three principal components is not sufficient to fully distinguish the classes. However, the PCA loadings plot shows the contribution of each Raman shift to the principal component, providing information about the Raman shifts that have a greater impact on the overall variation in the data (26). PC1 successfully captured the main variation patterns in the data (Fig. 2E). Significant positive and negative peaks were observed at wavelengths of 681 and 734 cm⁻¹, respectively. The positive peak at 681 cm⁻¹ is closely related to a specific vibrational mode of methionine, suggesting it may serve as a distinguishing feature for diagnosing BV (27, 28). PC2 captured the second major variation pattern orthogonal to PC1 (Fig. 2F), displaying a significant negative loading at 1,400 cm⁻¹, which is negatively correlated with the occurrence of BV. This wavelength may be considered to be *Lactobacillus* (29). The bacterial genus *Lactobacillus* is an important component of the vaginal microbiota, crucial for maintaining vaginal health.

### Characteristic peaks of SERS spectra

SERS spectroscopy has been extensively studied on biopolymers such as nucleic acids, proteins, lipids, and carbohydrates, and several studies have shown that the relationship between most Raman peaks and various molecular vibrations is interpretable, which can reveal differences between the chemical compositions of different samples (30). We described the chemical substances of BV-positive and BV-negative characteristic peaks by reviewing relevant literature, which is illustrated in Table 1. The positive characteristic peak at 1,143 cm^−1^ indicates vibrations within the C–N and C–C bond length distributions. The C–N stretching vibration corresponds to peptide bonds in proteins, while the C–C stretching vibration is related to the side chain or main chain structure of proteins (31). In BV-positive samples, the levels of porphyrin metabolites and specific amino acid catabolites (such as tryptamine in the tryptophan pathway) are significantly elevated, associated with the anaerobic bacterial activity characteristic of BV (28). The stretching vibration of the C = C double bond in the Raman spectra of tryptophan and porphyrin molecules is prominent, observed at 1,552 cm^−1^ (32). In tryptophan, the C = C stretching vibration reflects the local environment of the protein, especially its hydration state, while in porphyrin molecules, this vibration corresponds to the metal oxidation state. These changes highlight the distinct metabolic characteristics associated with BV, where amino acid degradation products are more prevalent, and intact amino acids are less abundant compared with BV-negative samples. Furthermore, the carboxyl stretch at 1,625 cm^−1^ generally pertains to carboxylic acid groups within proteins. This particular wavenumber suggests the carboxylic acid group is non-protonated, a characteristic consistent with higher pH or alkaline environments (33). When the vagina is alkaline, the production of anaerobic bacteria can lead to the typical “fishy odor” associated with BV infection (34). Meanwhile, the phospholipids at 1,100 cm^-1^ and carboxyl stretching vibration at 1,412 cm^−1^ in the BV-negative samples might characterize normal cellular membrane structure and function (35, 36).

**TABLE 1:** Significant characteristic peak components of BV-positive and BV-negative samples

BV status	Wavenumber (cm^−1^)	Band assignment	Reference
Positive	1,058	Triglycerides	(37)
	1,103	Phenylalanine	(38)
	1,143	C–N and C–C stretching in proteins	(31)
	1,370	Tryptophan	(39)
	1,517	C = C stretching	(40)
	1,552	C = C stretching of tryptophan and porphyrin	(32)
	1,625	Carboxyl stretch	(33)
Negative	1,100	Phospholipids	(35)
	1,412	Carboxyl stretching vibration	(36)
	1,560	Tryptophan	(41)

### OPLS-DA analysis of SERS spectra

**Fig 3:**
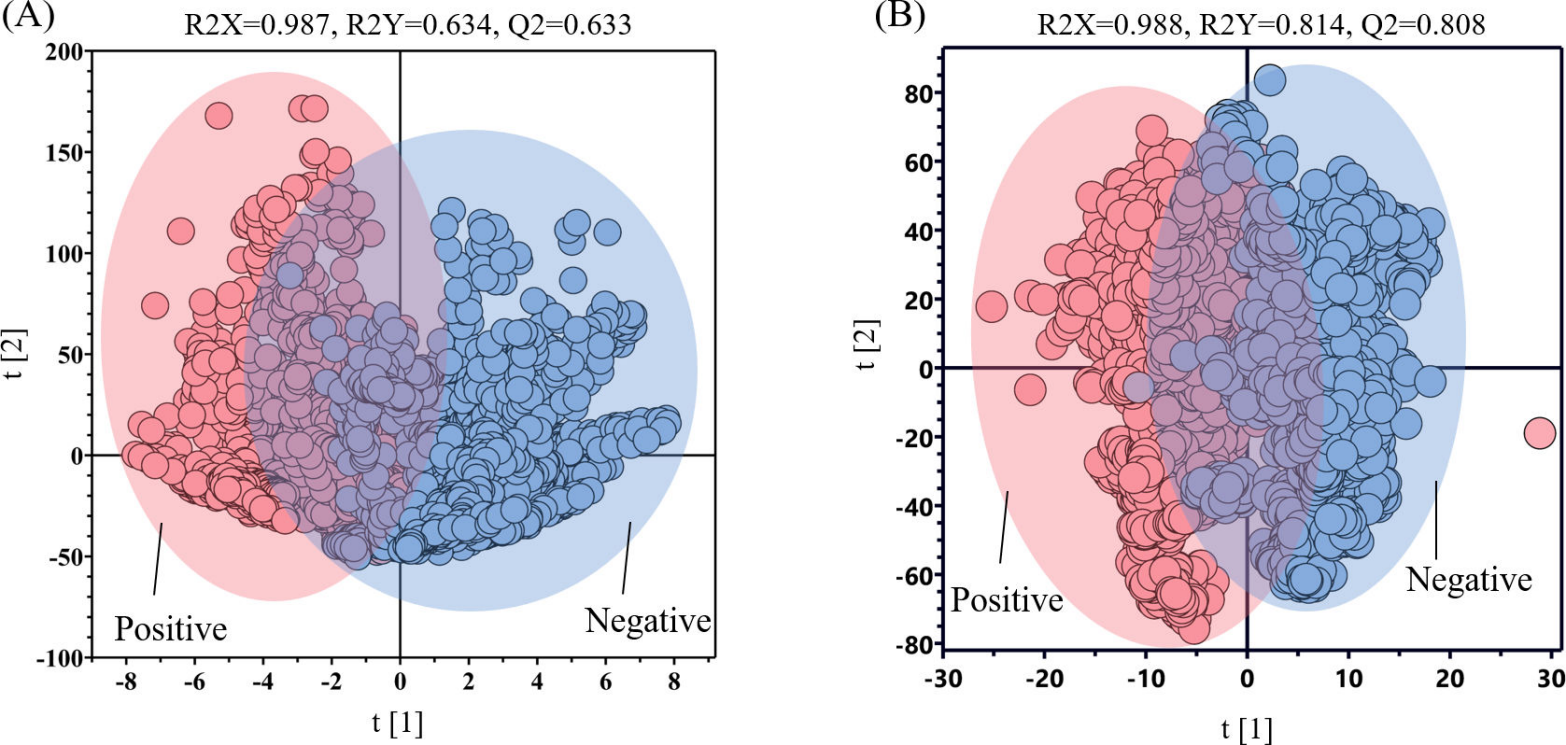
Comparison of OPLS-DA performance between the raw and normalized SERS data. (**A**) Raw and (**B**) normalized BV-positive and BV-negative scatter plots. R2X and R2Y represent the percentage of input matrix information that the model can explain respectively. Q2 is used to evaluate the predictive ability of the model. The range of three metrics is (0, 1), and the larger the value, the better the prediction performance of the model.

To determine the differences between BV-positive and BV-negative samples, we applied the orthogonal partial least squares discriminant analysis (OPLS-DA) for data analysis, which is illustrated in Figure 3. By comparing the raw (Fig. 3A) and normalized (Fig. 3B) SERS spectral data, we observed that normalization significantly enhanced sample separability. The R2X values for both data sets were close to 1 (0.987 and 0.988, respectively), indicating good data fitting. However, the predictive capability was notably better for the normalized data. The original data had an R2Y of 0.634 and a Q2 of 0.633, whereas the normalized data showed a marked improvement, with R2Y increasing to 0.814 and Q2 rising to 0.808. The classification performance of the model in OPLS-DA improves with increasing R2X and Q2 values. If the difference between these two indicators is less than or equal to 0.3, it indicates good performance (42). In this study, the difference between R2X and Q2 is 0.18, indicating a well-performing model.

### Comparison of supervised ML algorithms

We utilized CNN, SVM, and various ensemble learning models to analyze the optimized and preprocessed SERS spectral data. For each model, a series of search ranges for hyperparameters were predefined, and the *GridSearchCV* algorithm was employed to evaluate the performance of different hyperparameter combinations within the specified ranges, ultimately identifying the optimal hyperparameter configuration for each model (Table S1). The results demonstrate that (Table 2), under the optimal hyperparameter configuration, all models achieved a classification accuracy exceeding 0.90. Among these models, CNN performed the best, with scores on all evaluation metrics approaching 1. This indicates that the CNN model can handle the data set with high precision and efficiency. SVM excelled with over 97% metrics and 98.44% fivefold cross-validation, demonstrating strong generalization in classification. In comparison, GBoost and AdaBoost showed slightly lower performance, but still achieved over 93% in all metrics, indicating their potential application in classification tasks. However, the performance of DT was relatively poor, with a fivefold cross-validation result of only 88.33%, suggesting that the stability of the model needs improvement.

**TABLE 2:** Comparison of different ML algorithms

Algorithm	Accuracy	Precision	Recall	F1-score	Fivefold
CNN	99.97%	99.97%	99.89%	99.97%	99.90%
SVM	97.58%	97.58%	97.59%	97.58%	98.44%
RF	96.71%	96.71%	96.67%	96.71%	96.79%
XGBoost	95.83%	95.83%	95.84%	95.83%	95.00%
GBoost	94.46%	94.46%	96.51%	94.46%	95.95%
AdaBoost	93.27%	93.27%	93.26%	93.27%	93.19%
DT	92.92%	92.92%	92.94%	92.92%	88.33%

**Fig 4:**
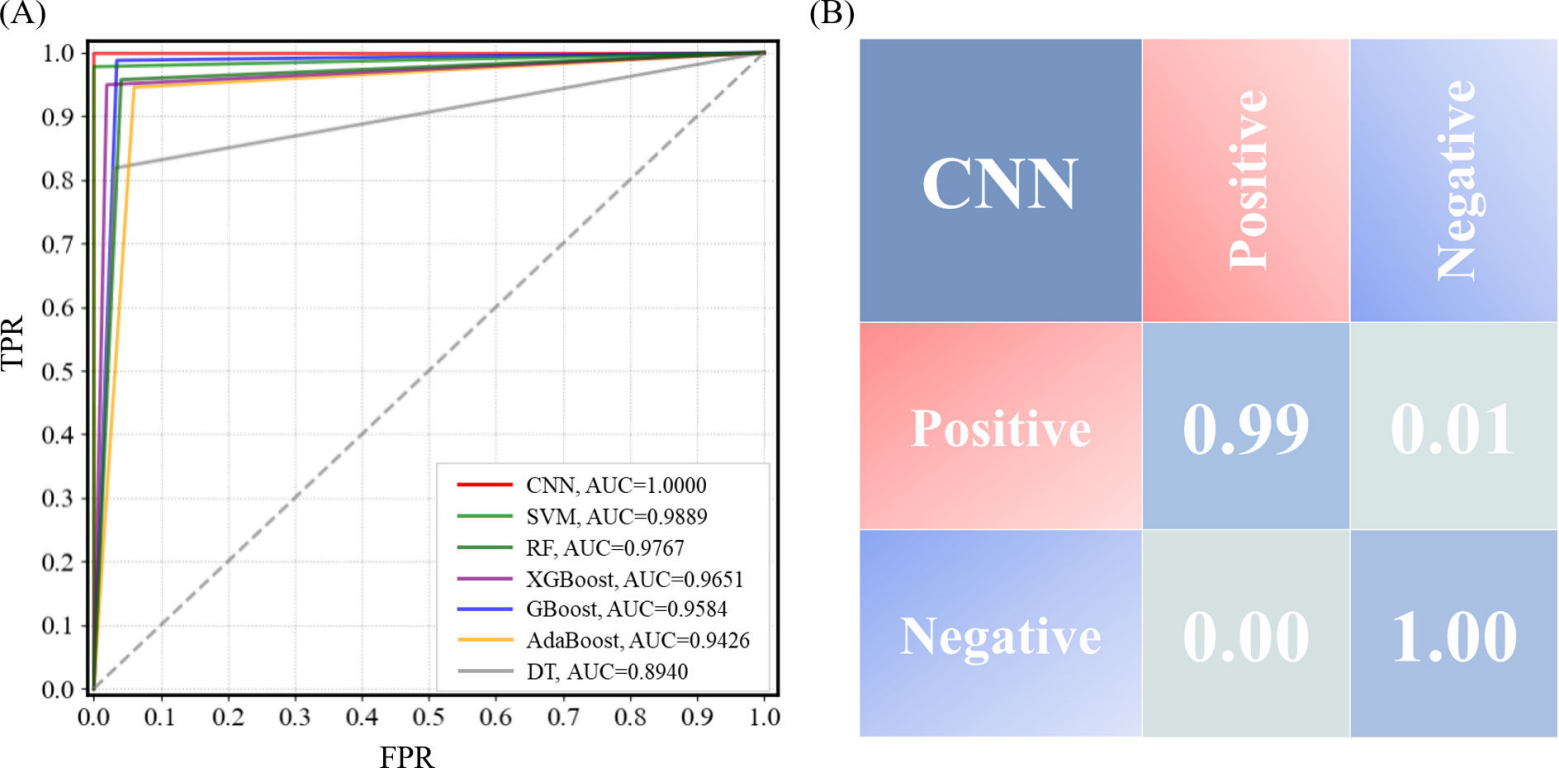
Performance evaluation of ML models. (**A**) ROC curves of seven algorithms and ROC curves of different colors have been quantified with AUC values. (**B**) Confusion matrix of CNN. TPR, true positive rate; FPR, false positive rate.

The specificity and sensitivity among the seven models were compared through receiver operating characteristic (ROC) curves, which is illustrated in Figure 4A. The model performance is better when the ROC curve is closer to the upper left corner. Upon examination of the ROC curve and AUC score, there are certain differences in the performance of different ML algorithms on this task. Due to its deep learning architecture, CNN is particularly adept at recognizing complex patterns. In this task, the CNN achieved a perfect AUC score of 1.00, indicating its excellent classification performance. We also plotted the learning curve of the CNN model to demonstrate the training process of the model (Fig. S2). SVM also performed strongly in classification tasks, although it may not capture complex patterns as effectively as CNN, its AUC score reached 0.9889. The AUC value for DT is 0.8940, the lowest among all models. To ensure the reliability and validity of the research results, we selected the best-performing CNN model for further study and clinical validation. Figure 4B represents the confusion matrix of the CNN, with an average accuracy of 99.5%.

### Interpretability of the deep learning model

**Fig 5:**
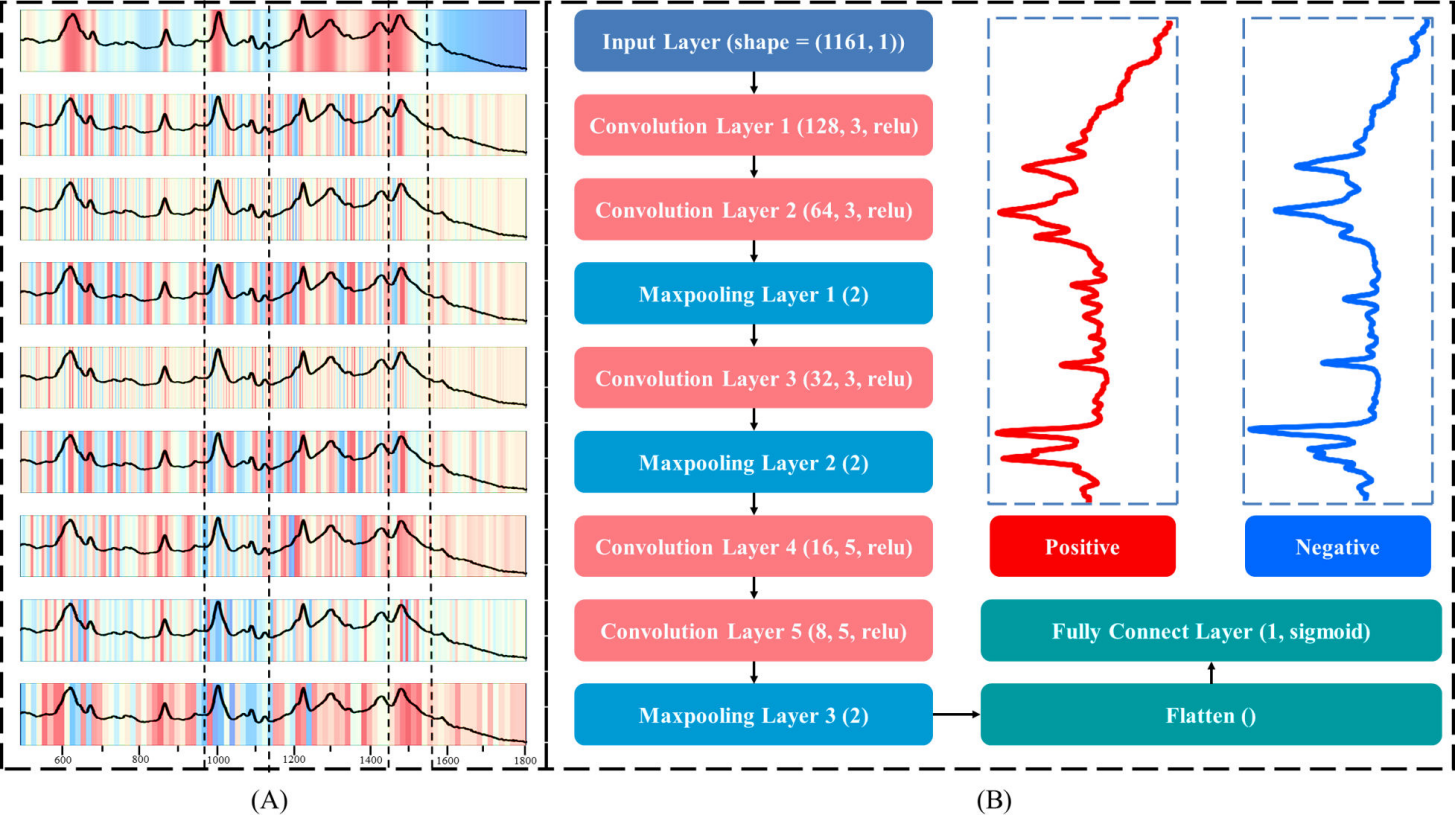
CNN architecture and classification activation mapping. (**A**) Heatmaps generated by Grad-CAM technology. (**B**) Structure diagram of CNN.

We used the Grad-CAM method to visualize the activation intensities of different layers of the CNN model when processing input data, which is illustrated in Figure 5A. This method helps to understand the specific features that the model focuses on during classification. Figure 5B shows the architecture of the CNN, illustrating how the data flow through the network and are ultimately classified as BV-positive or BV-negative samples. Through the Grad-CAM visualization method, it was observed that the model highly focuses on the Raman peak positions at 1,370, 1,517, 1,552, 1,560, and 1,625 cm^−1^ when classifying BV. In addition, the 1,560 cm^−1^ Raman peak serves as a unique identifier for BV-negative samples, while other specific peaks represent BV-positive samples. This demonstrates that the CNN model is capable of precisely identifying key Raman peaks during the classification of BV samples, particularly the 1,560 cm^−1^ Raman peak (41). There is a significant difference in the metabolism of this substance between BV-positive and BV-negative samples (28). During the model training process, the Raman shift range of 1,470–1,560 cm^−1^ was consistently a key area of focus. On the other hand, the model assigned a lower weight to the spectral features in the 990–1,110 cm^−1^ range, and it was also found in the loading plot that this range contributes almost nothing to sample differentiation. Initially, the model paid less attention to the 1,550–1,800 cm^−1^ range, but as the model continued to train, its points of focus evolved and adjusted. In distinguishing between BV-positive and BV-negative samples, the identification model is still able to capture significant differences between the spectra.

### Model validation analysis

**Fig 6:**
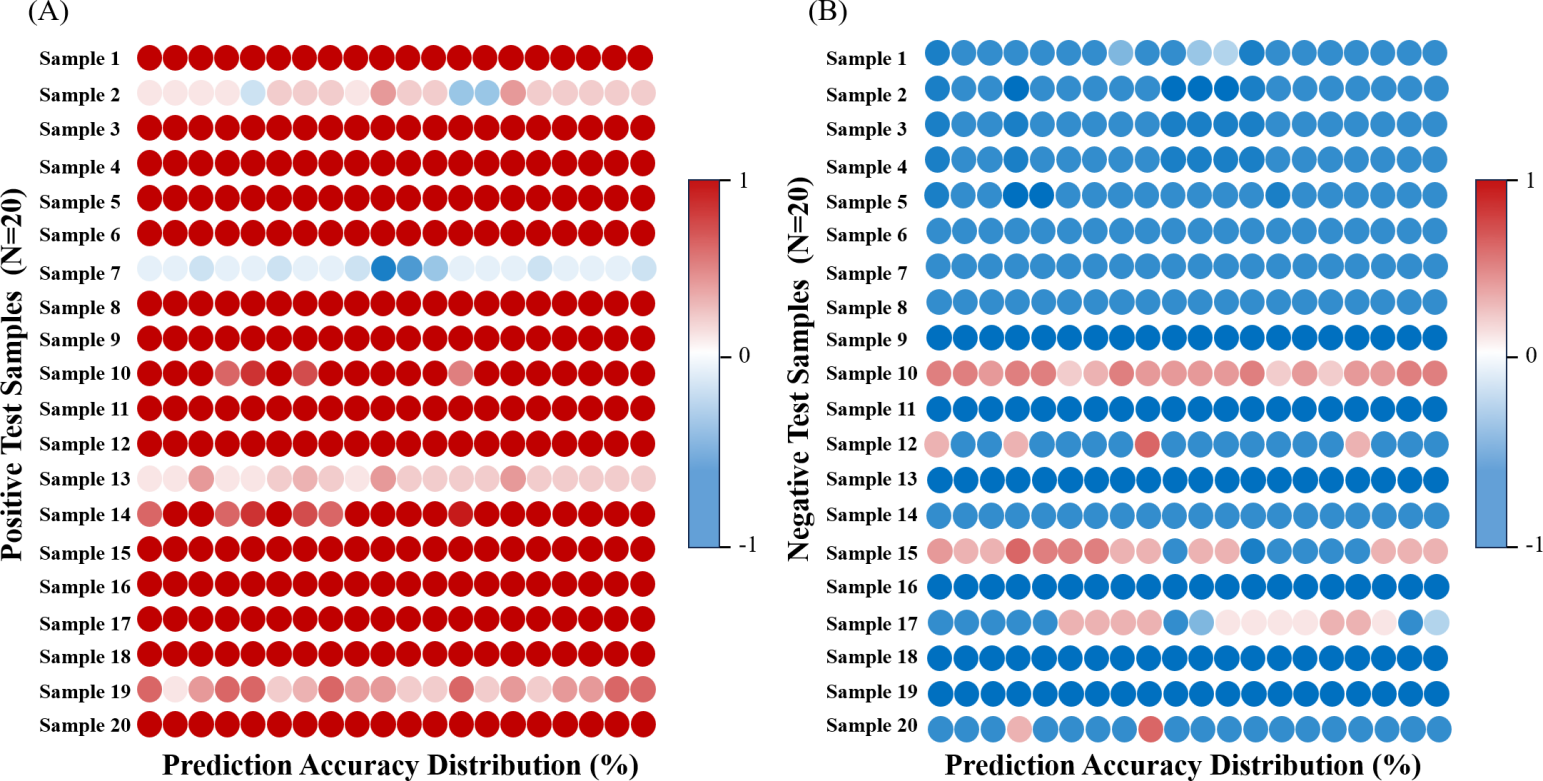
Blind sample testing of CNN models. (**A**) BV-positive prediction distribution. (**B**) BV-negative prediction distribution.

To validate the feasibility of the proposed method in a clinical setting, external independent validation was employed to test the performance of the model on unknown samples. We collected vaginal secretion samples from 40 BV patients (positive = 20, negative = 20) and generated 800 SERS spectra as test data. We then used a CNN model to analyze these data and compared the predictions with the actual clinical diagnostic outcomes. The average prediction accuracy for BV-positive patients was 90.75% (Fig. 6A), and for BV-negative patients, it was 87.25% (Fig. 6B). These results demonstrate the effectiveness and accuracy of our proposed method in real-world clinical settings. Additionally, the performance of the remaining seven ML algorithms on the external validation set is provided in Table S2.

## DISCUSSION

Although traditional BV detection methods, such as the Amsel criteria and Nugent scoring, are widely used in clinical practice, they often require significant manpower and time, and the operators need to have a certain level of expertise (3, 4). The rapid and accurate diagnosis of BV is of great importance for its intervention and treatment. SERS technology, due to its high specificity, sensitivity, and minimal sample preparation requirements, has been widely used for sample detection in biological samples (43). This study presents an efficient and low-cost BV detection method by combining deep learning models for automatic identification and data analysis, which has demonstrated potential for application in clinical environments.

Due to the complex composition of vaginal secretions, their spectral characteristics may contain multiple overlapping signals, which results in the SERS spectra of BV-positive and BV-negative samples exhibiting similarities in certain regions (44). Therefore, it is difficult to directly distinguish between these two states solely based on the average spectra. Taking into account such similarities, Pezzotti et al. used a linear polynomial expression of Gaussian-Lorentzian functions to match the experimental spectra based on the minimum scattering of the average Raman spectra, thereby generating deconvolution curves for a series of samples in the experiment (45). The experimental results showed that despite some morphological similarities between the SERS spectra of BV-positive and -negative samples, differences between them were successfully detected (Fig. 2A and B). For instance, the intensity of BV-positive samples at 681 cm^−1^ is significantly stronger than that of BV-negative samples. Similarly, we fitted peaks to the SERS spectra using the Vogit linear function, which revealed significant vibrational differences between BV-positive and -negative samples (Fig. 2C and D). However, the spectra contain an excessive number of variables (each representing a data point), which undoubtedly provides rich information but also increases the workload of data collection and the complexity of problem analysis. Therefore, a reasonable method is needed to reduce the number of original variables (dimensions) while preserving the important information contained in the original data set. Li et al. adopted the PCA method to achieve this goal by reducing dimensions (46). In this study, the PCA loadings analysis of the peak at 681 cm⁻¹ revealed that it has a significant contribution to the overall variation, representing methionine, which suggests that methionine metabolism may play an important role in the pathogenesis of BV. The research by Luo et al. showed that homocysteine (HCY), a key product of methionine metabolism, has a significantly positive correlation with BV, further supporting the potential role of methionine in BV (47). Compared to BV-positive samples, the SERS spectral analysis of BV-negative samples only revealed some normal membrane structural substances. These biomarker changes discovered through SERS spectroscopy, combined with existing clinical data research, can provide new insights for the early diagnosis and personalized treatment of BV, particularly through regulating methionine levels for the prevention and management of BV.

Despite our efforts to minimize potential interferences in Raman spectroscopy measurements during SERS signal acquisition, the collected spectral data inevitably contained non-target signals from experimental instruments or the samples themselves (48). To enhance the reliability and accuracy of the data, preprocessing becomes crucial. OPLS-DA is widely applied to the task of distinguishing SERS spectral data (49). To visualize the internal relationships between BV-positive and -negative samples, the OPLS-DA algorithm was employed for cluster analysis of BV spectral data (Fig. 3), utilizing R2X, R2Y, and Q2 evaluation metrics. The results indicate that after data preprocessing, the analytical performance of the spectral data is significantly improved. However, the OPLS-DA model relies excessively on training data, resulting in poor performance on new, unseen data (50). As it is primarily built based on data from known categories, this inherent limitation renders it insufficient when faced with complex data sets and unknown prediction tasks. Therefore, we have explored more advanced spectral data analysis methods.

In the field of BV diagnosis, Beck and Foster were the first to introduce ML algorithms but faced challenges in interpreting the features affecting the accuracy of the classification models (51). Subsequently, Jarvis et al. developed a new diagnostic tool based on a quantitative molecular diagnostic algorithm (CLS2.0q) for the diagnosis of BV, representing an initial attempt to combine ML algorithms with high-sensitivity detection technologies (52). In this study, we combined an interpretable ML model with a high-sensitivity SERS technique to broaden application scenarios. The normalized spectral data were used to build SVM, CNN, and five ensemble learning models. The selection of hyperparameters during model construction is crucial for classification performance. Even with high-quality data, inappropriate hyperparameter combinations can lead to suboptimal results. We used the highest-scoring parameter combination to test the SERS data of all samples and identify BV-positive and BV-negative samples in an independent data set. Among the seven models, the CNN showed the highest accuracy and efficiency in analyzing BV samples. This study employed the CNN model with the best performance for clinical validation. On the serum SERS data set of Cheng et al., the CNN model also performed well in detecting diseases, with a prediction accuracy of 97.78% on an independent test data set (53). This may be because SERS produces highly complex, high-dimensional spectral data and often contains local modes or peaks corresponding to specific molecular vibrations. In this case, CNN can capture low-level and high-level features that may be difficult to identify with traditional methods. Moreover, CNN is good at identifying local features through convolutional layers, so it is suitable for detecting subtle spectral features that are critical to accurate classification.

To address the black-box issue of explainability in deep learning models, Li et al. adopted Grad-CAM and other techniques to explain and visualize the decision-making process of neural networks (54). This study also adopted such a method to visualize the regional importance of CNN models. Among them, the Raman peak at 1,625 cm^−1^ plays a crucial role in the classification process. It corresponds to the carboxyl group of proteins, which is one of the essential functional groups of proteins involved in various biochemical reactions. Its changes may reflect the alterations in protein structure and function under BV conditions (55). Within the spectral range of 990–1,800 cm^−1^, the representative substance is primarily protein (56). Although the features within this range have a relatively low weight in the model, it does not imply that protein is unimportant in the identification of BV. This could be due to the model not yet fully learning the effective information from the protein features, or other features within this range (such as noise or other biomolecules) interfering with the model’s predictions. In inflammatory settings, specific proteins may degrade or be expelled from cells, resulting in the release of amino acids, such as tryptophan at 1,370 cm^−1^ and phenylalanine at 1,103 cm^−1^ (38, 39). As the main components of protein synthesis, the presence of these amino acids may be related to the breakdown and synthesis of proteins during cell damage and repair. In BV patients, these substances could indicate alterations to biochemical processes or an accumulation of bacterial metabolites. The Raman peak at 1,100 cm^−1^ corresponds to phospholipids (42). Phospholipids are crucial for maintaining cellular integrity and function, and changes in their structure and function may directly affect pathogen invasion and inflammatory responses in BV (57). The combination of Grad-CAM visualization technology further enhances the interpretability of the CNN model, highlighting key spectral features related to BV identification. Although the CNN achieved the highest accuracy in predicting the infection status of BV samples, with an accuracy rate close to 1, we aimed to enhance the credibility of this “perfect” prediction result by extending the model to practical clinical environments. In this study, we collected 40 BV samples with unknown infection status from clinical settings and analyzed their BV SERS spectra. By comparing the results with the actual infection status based on clinical microscopy, the results showed that the CNN achieved an accuracy rate of 89% on the blind sample data set, indicating that the model can accurately and effectively predict the infection status of BV.

Despite the promising results, our study has several limitations. The sample size, although sufficient for preliminary validation, is relatively small and may not fully represent the variability present in a broader clinical population. Future studies should investigate larger and more diverse cohorts to validate our findings and enhance the generalizability of the model. Additionally, in complex biological systems, factors such as mixed cell types and metabolic diversity may obscure or mask sample variations, thereby weakening the analytical capability of SERS (58). Therefore, more advanced sample preparation and analysis techniques are needed for practical clinical applications to improve detection specificity. Research also needs to pay special attention to the fairness of models across different racial groups. Existing studies have shown that the performance of ML models in diagnosing BV can vary significantly among different races (59). Therefore, ensuring the diversity of the data set to include samples from multiple racial groups is crucial to avoid model bias toward specific races and is an important direction for future research.

In conclusion, our study demonstrated that the combination of SERS and ML techniques can accurately diagnose BV. This automated diagnostic system shows the potential for widespread use in clinical point-of-care settings, especially for primary care clinics and community hospitals.

## MATERIALS AND METHODS

### Chemical materials and instruments

Silver nitrate (AgNO₃) and sodium citrate dihydrate (C₆H₉Na₃O₉) were purchased from SinoPharm Chemical Reagent Co., Ltd. (Shanghai, China). The BVBlue Bacterial Vaginosis Detection Kit (Sialidase), which contains all the test solutions including potassium acetate buffer (0.5 mg/mL, pH 5.3–6.2) and chromogenic solution (NaOH solution, 40.0 mg/mL, pH 11.0–13.0), was purchased from Beijing Mingwude Biotechnology Co., Ltd. (Beijing, China). Gram staining solution was purchased from Baso Biotechnology Co., Ltd. (Zhuhai, China). Single-side polished silicon wafers were procured from Hangzhou Hangdan Optoelectronic Technology Co., Ltd. (Hangzhou, China). The optical microscope used for sample examination was purchased from Olympus Corporation (Japan). The inVia Raman microscope (Renishaw Plc., New Mills, Wotton-under-Edge, UK) was used to collect Raman signals.

### BV sample collection

In this study, we collected 100 vaginal fluid samples (50 negative samples and 50 positive samples) from outpatients at the Guangdong Provincial People’s Hospital in China (baseline information is provided in Table S3). Additionally, we used 40 vaginal fluid samples for blind testing to assess the model’s accuracy (baseline information is provided in Table S4). The inclusion criteria for the samples were (i) female patients aged between 18 and 65 years, excluding those with human papillomavirus (HPV) infections; (ii) the samples should come from individuals potentially undergoing bacterial vaginosis screening, including patients with symptoms such as vaginal itching and abnormal discharge, as well as those without related clinical symptoms; and (iii) the samples meeting the above characteristics were selected as inclusion samples, which should have sufficient quantity, clearly traceable sources, and varied factors. The collection and handling of the samples complied with practical manuals or relevant regulations. The related information for the samples was complete, including age, gender, and clinical diagnosis.

### BVBlue Test combined with clinical microscopy to diagnose BV

Collect vaginal discharge from the patient using a sterile swab and observe whether the discharge is homogenous and thin. Evenly smear the vaginal discharge sample on a glass slide (dry smear) to create a thin film, and perform Gram staining. The staining steps include fixing the sample, staining, washing, and drying. After staining, observe under a microscope at 1,000× magnification (oil immersion), ensuring proper use of immersion oil. Observe and record the number of clue cells and changes in the bacterial community, particularly the presence of lactobacilli and other bacteria (such as *Gardnerella* and anaerobes). Next, use the wet mount method to observe the collected sample. Place the vaginal discharge sample on a glass slide, add a drop of saline, mix gently, and cover with a coverslip. Observe under a microscope at 400× magnification, looking for and recording the presence of clue cells. Clue cells are vaginal epithelial cells covered with bacteria, with indistinct edges and a granular appearance. Then, perform the BVBlue Test. Immerse the sterile swab in the BVBlue detection solution, and gently rotate the swab to release the sample as much as possible. Place the swab and detection solution together in an incubator at 37°C for 10 minutes, ensuring the detection solution completely covers the swab. After incubation, add 1–2 drops of developer solution and gently shake. Observe the final result after 3 minutes: blue indicates bacterial vaginosis, and yellow indicates a negative result. Finally, combine the BVBlue Test results with the microscopic observations. The proportion of clue cells among vaginal epithelial cells is an important indicator for diagnosing bacterial vaginosis. If both the BVBlue Test and microscopic observations are positive, bacterial vaginosis is confirmed. This method complies with the general requirements of the 2018 European International Federation for Sexually Transmitted Diseases (STD) Control/World Health Organization (WHO) Guidelines for the Management of Vaginal Discharge (Vaginitis) (9).

### SERS spectral generation

The following procedure was used to prepare a negatively charged silver nanoparticle substrate (60). In a sterile container, weigh 33.72 mg of silver nitrate and dissolve it in 200 mL of deionized water. Mix and heat on a magnetic stirrer. After boiling, add 8 mL of sodium citrate solution (1 wt%) and stir at 650 r/min for 40 minutes. Stop heating, allow to cool naturally to room temperature, and mix thoroughly. Transfer 1 mL of the solution to a sterile Eppendorf (EP) tube and centrifuge at 7,000 r/min for 7 minutes. Discard the supernatant and resuspend the pellet in 100 µL of deionized water to obtain the desired silver nanoparticles. A 0.5 mg/mL IBX-4041 solution with a pH between 5.3 and 6.2 was prepared using potassium acetate buffer. The collected specimen is then inoculated into the IBX-4041 solution. Take 2.5 µL of IBX-4041 solution and 2.5 µL of silver nanoparticles, mix well, and spot onto a silicon wafer to obtain high-sensitivity Raman signal detection signals for BV-positive and BV-negative samples. The BV-positive and BV-negative samples were detected using the inVia Raman microscope. The parameters for the dried spots testing were set as follows: excitation wavelength of 785 nm, laser power of 10 mW, aperture slit of 50 µm, grating of 1,200 lines/mm, spectral resolution of 4.7–8.6 cm^−1^, and detection spectral range of 500–1,800 cm^−1^. Before spectral acquisition, the Raman peak of the silicon wafer at 520 cm^−1^ was used for wavenumber calibration. Dark current was subtracted from the spectrum during the integration time. For each sample point, 50 loci were randomly selected for SERS signal detection.

### Average SERS spectra and deconvolution analysis

After acquiring the SERS spectral data, calculations and analyses were performed to average the spectral data within the SERS spectral range between 500 and 1,800 cm^−1^. The average Raman intensities were calculated at the same Raman shifts for all SERS spectral data for both BV-positive and BV-negative samples. The 20% SD band was visualized as a shaded area around the average SERS spectrum using Origin software (OriginLab, USA). The error band’s width indicates the SERS spectrum’s reproducibility: a wider band means poorer reproducibility. The deconvolution operation was then conducted on each average SERS spectrum to delve into the spectral characteristics. The *fit_peaks_pro* function in the Origin software was used to fit the peaks, employing the maximum value and half-width of the *Voigt* function to deduce the Doppler and Lorentz lines, thereby resolving the spectrum’s deconvoluted sub-bands. The fitting process was repeated until convergence was achieved. For all characteristic peaks identified by deconvolution, chemical substances and molecular vibrations corresponding to these peaks were selected through a comprehensive literature review. In addition, we obtained the principal component loadings of the data to identify the contribution of each variable to the principal component. The decomposition module was imported from the scikit-learn library, and a PCA function was created, specifying the number of principal components to be extracted as 2. A set of loading vectors named “loadings” were extracted while the components of the PCA function were assessed. These loading vectors were unit vectors, with their directions representing the projection of the original variables in the principal component space, and their magnitudes indicating the weights of the projections (61).

### Clustering analysis of SERS spectra of BV-negative and BV-positive samples

For clustering analysis, we used the OPLS-DA algorithm to discriminate the SERS spectra of BV-negative and BV-positive vaginal fluids. Before implementation, SERS spectral data (a total of 5,000 spectra, with 50 spectra per sample from 100 samples) were individually imported into the Unscrambler X 10.4 (64-bit) software for maximum normalization to eliminate the dimensional impact of the data. Both raw and normalized SERS spectral data were then imported into the SIMCA software (Umetrics, Sweden). 0 and 1 were used to represent the BV-positive and BV-negative spectra. These numbers were placed in a special column, designated as the label column, to indicate the BV status of the corresponding vaginal fluids. The software automatically determined the optimal number of components for the model. After the model was trained, the optional *Scatter* was selected in the *Score* function to visualize the model’s classification results via scatter plots. The clustering results were evaluated using the R2X, R2Y, and Q2 indices. In specificity, R2X measures the model’s ability to explain the input data, R2Y assesses the model’s fit to the classification variable, and Q2 reflects the predictive reliability of the model and its performance on unseen data, which served as indicators to evaluate the robustness and predictability of the clustering analysis (62).

### Construction of supervised machine learning models

To construct a model that can effectively predict the BV, we applied seven ML algorithms to the SERS spectral data: AdaBoost, DT, GBoost, RF, SVM, XGBoost, and CNN. In specificity, the spectral data were put into the input layer and the input_shape was set to (1,161, 1). The spectral data are a two-dimensional matrix, where 1,161 represents the number of wavelength points of the spectral data and 1 represents the single channel data of each wavelength point. The data set was then divided into training, validation, and test data sets according to the ratio of 6:2:2 through the *train_test_split* function. The training and validation data sets were used to train and verify the model, while the test set serves as a data set independent of the training and validation data sets. The *GridSearchCV* function was utilized to optimize parameters for six ML algorithms to identify the optimal model configuration. We used three convolutional layers after the input layer for the CNN model to process and extract features within the SERS spectral data. After each convolutional layer, nonlinearity was added to the output of the convolutional layer through the rectified linear unit (ReLU) activation function, improving the fitting ability and the complexity of feature capture. The number of filters was set to 8, 8, and 16, respectively. In the max pooling layer, *pool_size* is set to 3, which could reduce the dimensionality and capture more important features. Before connecting the fully connected layer, the abstract features extracted from the previous layers were flattened into one-dimensional vectors using the flattened layer and were provided to the fully connected layer for further processing. The last fully connected layer in the network used a dense layer with a sigmoid activation function designed to handle binary classification problems.

### Model performance evaluation and interpretive analysis

This study employed six key metrics to evaluate the performance of the classification model such as accuracy, precision, recall, F1-score, fivefold cross-validation, and model fitting time. An independent test data set, separate from the training set, was used to validate the model, with a particular focus on true-positive and false-positive outcomes. In the context of feature performance evaluation, accuracy was computed using the *accuracy_score* function, which represented the proportion of correctly predicted samples relative to the total variety of positive and negative samples. The *precision_score* function was used to determine the precision, which signified the proportion of correctly predicted positive instances against all predicted positive instances. The *recall_score* function was utilized to measure recall, which denoted the proportion of correctly predicted positive instances against all actual positives. While precision and recall served as a balanced pair of evaluation metrics, the F1-score served as their harmonious average, providing a comprehensive reflection of the model’s overall performance in terms of classification quality. The harmonious performance of these two indicators was taken into account using the *f1_score*. To gauge the model’s generalization capacity, the *cross_val_score* function was implemented. The model efficiency was noteworthy in practical applications, so the time function was employed to calculate the model’s fitting time to maximize efficiency while minimizing computing resources. Additional evaluation techniques included the utilization of the *roc_curve* function to compute the ROC curve for each category and visually inspect its AUC value. The construction of a confusion matrix through the *confusion_matrix* function allows the examination of the sample distribution per category in the model’s predictive outcomes.

### Interpretive analysis of SERS spectra

To gain a deeper understanding of the internal mechanisms behind the classification decisions made by the CNN model, we employed the Grad-CAM algorithm. Grad-CAM serves as a method for visualizing the CNN decision-making process, highlighting the most critical regions in an image for predicting a specific class. Initially, we constructed a new model *grad_model*, which retained the original model’s weights but included the activation map of the target convolutional layer (layer_name) and the final prediction results at the output end. This approach enabled us to capture feature information from intermediate layers and the class predictions simultaneously. Subsequently, we utilized the *tf.GradientTape* function in TensorFlow, which had the functionality to record gradient information for the activation map of the target layer relative to the prediction of a specific class. Specifically, we obtained prediction probabilities through forward propagation, extracted the class with the highest prediction probability (i.e., the most likely class according to the model), and then calculated the gradients of this class relative to the activation map of the target layer. These gradients reflected the degree of contribution made by each feature map in the target layer toward the prediction of that particular class. We performed global average pooling on these gradients to obtain a concise representation, resulting in a weight vector. This weight vector signified the importance of each feature map in the target layer for the specific class. Finally, we multiplied the weight vector by the activation map of the target layer and then averaged the outcome along the feature map channel direction, generating a two-dimensional heatmap. This heatmap intuitively showcased the regions within the input image that exerted the greatest influence on the prediction of a specific class, thereby facilitating a better understanding of the decision-making process.

### Independent validation of model performance

To ensure the robustness of the model, its performance was assessed by predicting the infection status of a group of newly collected vaginal fluid samples without any pre-existing knowledge of their actual conditions. Each sample was observed by a trained specialist using a microscope in the clinical laboratory of Guangdong Provincial People’s Hospital. All samples were also tested using BVBlue Test. The combined results were used to determine the genuine infectious status of the vaginal fluids. The samples were subjected to Raman spectroscopy following laboratory assessments. The generated SERS spectra were input into the optimized CNN model for prediction. To ensure that the experiment was conducted fairly and unbiasedly, no knowledge of the actual infectious status of the samples was known. After obtaining the prediction results of the model, they were compared with the results from the clinical laboratory to determine the prediction accuracy of the CNN model.

## Data Availability

We have uploaded the raw data and key code to the GitHub website at https://github.com/Wennie5/BV-rapid-detection/tree/master.

## SUPPLEMENTAL MATERIAL

Supplemental material msystems.01058-24-s0001.docx Fig. S1 and S2; Tables S1 to S4.

ASM does not own the copyrights to Supplemental Material that may be linked to, or accessed through, an article. The authors have granted ASM a non-exclusive, world-wide license to publish the Supplemental Material files. Please contact the corresponding author directly for reuse.
