# Supplementary material for: Rapid diagnosis of bacterial vaginosis using machine-learning-assisted surface-enhanced Raman spectroscopy of human vaginal fluids
Source: mSystems. 2024 Dec 10;10(1):e01058-24. doi: 10.1128/msystems.01058-24 (PMC11748538; doi:10.1128/msystems.01058-24)
Supplement: Supplemental material — Fig. S1 and S2; Tables S1 to S4. [file msystems.01058-24-s0001.docx]

**Supplementary Material**


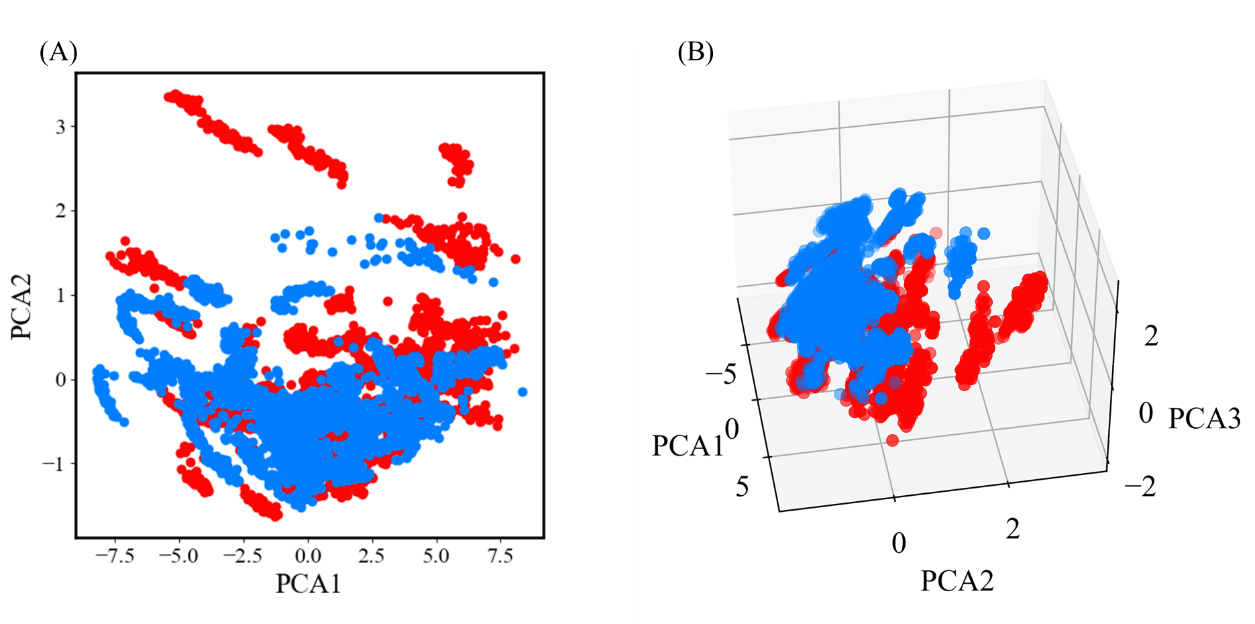


**Supplementary Figure S1** Two-dimensional and three-dimensional PCA analysis of BV’s negative and positive SERS spectra. (A) Two principal components of PCA analysis. (B) Three principal components of PCA analysis. Note: We have plotted the PCA scatter plots in 2D and 3D. The separation effect in 2D and 3D plots is not obvious, indicating that the variance explained by the first three principal components cannot distinguish different classes fully. We will store this result in the attachment for readers' reference. However, the loading plot shows the contribution of each variable (Raman shift) to the principal component. The graphs in this article reveal which Raman shifts are most important in PCA and which signals contribute the most to PC1 and PC2. So, even if the clustering effect is not ideal, the PCA loading plot can still provide valuable information about which Raman shifts have the greatest impact on the overall variation of the data.


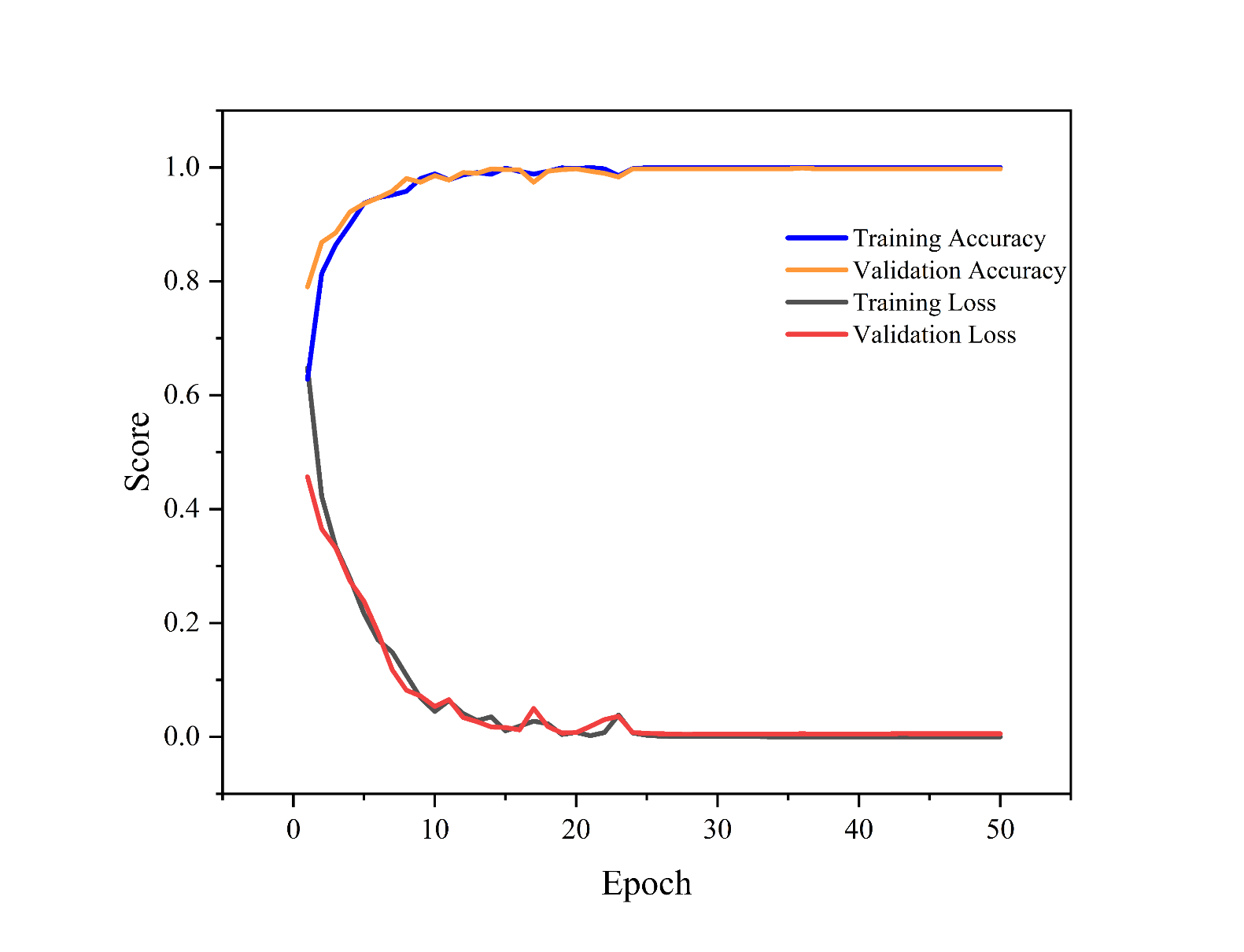


**Supplementary Figure S2** CNN Learning Curve. Training and validation loss and accuracy curves during model training. Note: The learning curve of the CNN model shows that the training accuracy and loss changed significantly during the first 10 epochs and began to plateau around epoch 20. The training and validation accuracies were nearly identical, indicating the model has good predictive ability for unknown data. The training loss was slightly lower than the validation loss, but the difference was minimal, suggesting that the model did not exhibit significant overfitting. Overall, the learning curve demonstrates the robustness of the CNN model on the dataset used in this study.

**Supplementary Table S1** The Best Combination of hyperparameter for Different Machine Learning Algorithms

| **Algorithms** | **Parameter Range** | **Optimum Parameter** |
| --- | --- | --- |
| **AdaBoost** | **learning_rate** = [0.1, 1, 0.01, 0.001,0.0001],  **n_estimators** = [50, 60, 70, 80, 90, 100, 110, 120, 130, 140, 150, 160, 170, 180] | **learning_rate** = 1, **n_estimators** = 170 |
| **Decision Tree** | **criterion** = ['gini', 'entropy'],  **max_depth** = range (1, 30), **max_features** = [21, 22, 23, 24, 25, 26, 28, 29, 30, 'auto'] | **criterion** = 'gini',  **max_depth** = 25,  **max_features** = 23 |
| **GBoost** | **n_estimators =** [120, 130, 140, 145, 150, 160, 170, 175, 180, 185],  **learning_r =** [0.1, 1, 0.01, 0.001] | **learning_rate** = 1, **n_estimators** = 170 |
| **Random Forest** | **Criterion** = ['gini', 'entropy'],  **max_depth** = range (1, 10),  **n_estimators** = [50, 60, 70, 80, 90, 100, 110, 120, 130, 140, 150, 160, 170, 180] | **criterion** = 'gini', **max_depth** = 9, **n_estimators** = 145 |
| **SVM** | **Cs =** [0.0001, 0.001, 0.01, 0.1, 1, 2, 3, 4, 5, 10],  **gamma =** [0.0001, 0.001, 0.01, 0.1, 1],  kernel = ['rbf', ' linear '] | **Cs =** 1,  **gamma =** 0.0001,  **kernel =** 'linear',  **probability =** True |
| **XGBoost** | **n_estimators =** [50, 60, 70, 80, 90, 100, 110, 120, 130, 140, 150, 160, 170, 180],  **learning_r =** [0.1, 1, 0.01, 0.001, 0.0001] | **learning_rate** = 0.1, **n_estimators** = 150 |

**Supplementary Table S2** Comparison of different ML algorithms on an independent validation set.

| **Algorithm** | **Accuracy** | **Precision** | **Recall** | **F1-score** |
| --- | --- | --- | --- | --- |
| CNN | 87.20% | 96.38% | 77.30% | 85.79% |
| SVM | 81.75% | 100.00% | 63.50% | 77.68% |
| DT | 79.70% | 72.71% | 95.10% | 82.41% |
| XGBoost | 67.45% | 60.61% | 99.70% | 75.39% |
| AdaBoost | 61.35% | 56.49% | 98.80% | 71.88% |
| RF | 52.10% | 51.08% | 99.60% | 67.53% |
| GBoost | 46.10% | 47.95% | 91.20% | 62.85% |

Note: We tested the model on an external independent validation set. Although the performance of the CNN model declined on the external validation set, it remained the best-performing model among all, with a classification accuracy of 87.20%. However, the performance of the remaining models significantly declined in the external cohort due to being trapped in local optima during the training process and the occurrence of overfitting.

**Supplementary Table S3** Baseline information of the population for model training.

| **No.** | **Age** | **BV Results** | **BVBlue** | **Epithelial Cells** | **Clue Cell** | **Gram-Negative C*occi*** | **Gram-Positive *Cocci*** | **Gram-Positive *Bacilli*** | **Gram- Positive Short *Bacilli*** | **Gram- Negative Bacteria** | **Corynebacterium** |
| --- | --- | --- | --- | --- | --- | --- | --- | --- | --- | --- | --- |
| N9.6-2 | 39 | negative | negative | 1/2 field of view | 0 | - | - | 2+ | - | - | - |
| N9.6-4 | 34 | negative | negative | 1/2 field of view | 0 | - | - | - | 1+ | - | - |
| N9.6-5 | 36 | negative | negative | 1/2 field of view | 0 | - | - | 4+ | - | - | - |
| N9.6-6 | 48 | negative | negative | Full field of view | 0 | - | ± | 1+ | 2+ | - | - |
| N9.6-9 | 27 | negative | negative | Full field of view | 0 | - | - | - | 4+ | - | - |
| N9.6-11 | 48 | negative | negative | 1/2 field of view | 0 | - | - | 1+ | - | - | - |
| N9.6-12 | 28 | negative | negative | 1/2 field of view | 0 | - | - | 3+ | - | - | - |
| N9.6-13 | 31 | negative | negative | 1/2 field of view | 0 | - | 1+ | 1+ | 2+ | - | - |
| N9.6-14 | 45 | negative | negative | Full field of view | 0 | - | - | 4+ | - | - | - |
| N9.6-15 | 62 | negative | negative | 1/2 field of view | 0 | - | - | 1+ | - | - | - |
| N9.6-18 | 43 | negative | negative | Full field of view | 0 | - | - | 4+ | - | - | - |
| N9.6-19 | 60 | negative | negative | a small amount | 0 | - | - | - | 1+ | - | - |
| N9.6-20 | 22 | negative | negative | Full field of view | 0 | - | 1+ | - | 4+ | - | - |
| N9.6-21 | 39 | negative | negative | 1/2 field of view | 0 | - | ± | 1+ | 3+ | - | - |
| N9.6-22 | 49 | negative | negative | a small amount | 0 | - | ± | - | 1+ | - | - |
| N9.6-23 | 29 | negative | negative | 1/2 field of view | 0 | - | 1+ | - | 4+ | - | - |
| N9.6-24 | 59 | negative | negative | Full field of view | 0 | - | - | - | 3+ | - | - |
| N9.6-25 | 50 | negative | negative | a small amount | 0 | - | 1+ | - | - | - | - |
| N9.6-26 | 22 | negative | negative | 1/2 field of view | 0 | - | - | - | 2+ | - | - |
| N9.6-27 | 27 | negative | negative | Full field of view | 0 | - | - | - | - | - | 3+ |
| N9.6-28 | 34 | negative | negative | 1/2 field of view | 0 | - | - | 4+ | - | - | - |
| N9.6-29 | 26 | negative | negative | Full field of view | 0 | - | - | 4+ | - | - | - |
| N9.6-30 | 54 | negative | negative | a small amount | 0 | - | - | ± | - | - | - |
| N9.6-31 | 28 | negative | negative | Full field of view | 0 | - | - | 3+ | - | - | - |
| N9.6-32 | 35 | negative | negative | Full field of view | 0 | - | - | 3+ | - | - | - |
| N9.6-33 | 37 | negative | negative | 1/2 field of view | 0 | - | 1+ | 1+ | 2+ | - | - |
| N9.6-34 | 30 | negative | negative | 1/2 field of view | 0 | - | 2+ | 1+ | - | - | - |
| N9.6-35 | 32 | negative | negative | Full field of view | 0 | - | 1+ | - | 3+ | - | - |
| N9.6-38 | 40 | negative | negative | Full field of view | 0 | - | - | - | 4+ | - | - |
| N9.6-39 | 32 | negative | negative | 1/2 field of view | 0 | - | 1+ | 1+ | 1+ | - | - |
| N9.6-40 | 25 | negative | negative | 1/2 field of view | 0 | - | 1+ | 3+ | - | - | - |
| N9.6-42 | 39 | negative | negative | 1/2 field of view | 0 | - | 1+ | 1+ | - | - | - |
| N9.6-43 | 31 | negative | negative | 1/2 field of view | 0 | - | - | 3+ | - | - | - |
| N9.6-44 | 24 | negative | negative | 1/2 field of view | 0 | - | - | 4+ | - | - | - |
| N9.6-46 | 52 | negative | negative | 1/2 field of view | 0 | - | 1+ | 2+ | 2+ | - | - |
| N9.6-48 | 41 | negative | negative | Full field of view | 0 | - | 1+ | - | 4+ | - | - |
| N9.6-49 | 39 | negative | negative | Full field of view | 0 | - | - | 3+ | - | - | - |
| N9.6-60 | 36 | negative | negative | Full field of view | 0 | - | - | 3+ | - | - | - |
| N9.6-62 | 45 | negative | negative | Full field of view | 0 | - | 1+ | - | 3+ | - | - |
| N9.6-63 | 36 | negative | negative | Full field of view | 0 | - | - | 3+ | - | - | - |
| N9.6-65 | 24 | negative | negative | Full field of view | 0 | - | 1+ | - | 4+ | - | - |
| N9.6-601 | 42 | negative | negative | 1/2 field of view | 0 | - | - | 4+ | - | - | - |
| N9.6-73 | 65 | negative | negative | 1/2 field of view | - | - | - | - | - | - | 2+ |
| N9.6-74 | 34 | negative | negative | 1/2 field of view | - | - | - | 4+ | - | - | - |
| N9.6-75 | 44 | negative | negative | Full field of view | - | - | - | 4+ | - | - | - |
| N9.6-76 | 28 | negative | negative | 1/2 field of view | - | - | 1+ | - | 3+ | - | - |
| N9.6-77 | 31 | negative | negative | 1/2 field of view | - | - | - | 2+ | 1+ | - | - |
| N9.6-78 | 31 | negative | negative | 1/2 field of view | - | - | 1+ | - | 2+ | - | - |
| N9.6-79 | 29 | negative | negative | 1/2 field of view | - | - | - | - | 3+ | - | - |
| N10.7-70 | 40 | negative | negative | 1/2 field of view | - | - | 1+ | - | 4+ | - | - |
| P9.6-3 | 57 | positive | positive | Full field of view | 0 | - | 1+ | - | 4+ | - | - |
| P9.6-37 | 24 | positive | positive | 1/2 field of view | 0 | - | 1+ | - | 4+ | - | - |
| P9.6-45 | 43 | positive | positive | Full field of view | 0 | - | 1+ | - | 4+ | - | - |
| P9.6-603 | 37 | positive | positive | 1/2 field of view | - | - | 1+ | - | 4+ | - | - |
| P9.7-80 | 53 | positive | positive | a small amount | - | - | 1+ | - | 2+ | - | - |
| P9.7-36 | 47 | positive | positive | Full field of view | - | - | 1+ | 1+ | 4+ | - | - |
| P9.7-40 | 35 | positive | positive | Full field of view | - | - | 1+ | - | 4+ | - | - |
| P9.7-44 | 35 | positive | positive | Full field of view | find | - | 1+ | - | 4+ | - | - |
| P9.8-4 | 37 | positive | positive | 1/2 field of view | - | - | 1+ | - | 3+ | - | - |
| P9.8-20 | 33 | positive | positive | 1/2 field of view | - | - | 1+ | - | 3+ | - | - |
| P9.8-28 | 47 | positive | positive | Full field of view | - | - | 1+ | - | 3+ | - | - |
| P9.19-18 | 49 | positive | positive | Full field of view | - | - | 1+ | - | 4+ | - | - |
| P9.19-41 | 27 | positive | positive | 1/2 field of view | - | - | 1+ | - | 3+ | - | 1+ |
| P9.19-49 | 32 | positive | positive | Full field of view | - | - | 1+ | 1+ | 4+ | - | - |
| P9.19-605 | 40 | positive | positive | 1/2 field of view | - | - | 1+ | - | 4+ | - | - |
| P9.19-608 | 67 | positive | positive | 1/2 field of view | - | - | 1+ | - | 2+ | - | - |
| P9.20-34 | 36 | positive | positive | 1/2 field of view | - | - | 1+ | - | 4+ | - | - |
| P9.20-39 | 49 | positive | positive | Full field of view | - | - | - | - | - | - | 4+ |
| P9.20-49 | 46 | positive | positive | 1/2 field of view | - | - | 1+ | - | 3+ | - | - |
| P9.20-51 | 24 | positive | positive | 1/2 field of view | - | - | - | - | 4+ | - | - |
| P9.20-602 | 49 | positive | positive | Full field of view | find | - | 1+ | - | 4+ | - | - |
| P9.20-620 | 34 | positive | positive | Full field of view | - | - | 1+ | - | 4+ | - | - |
| P9.21-9 | 45 | positive | positive | Full field of view | - | - | 1+ | - | 3+ | - | - |
| P9.21-31 | 32 | positive | positive | 1/2 field of view | - | - | 1+ | - | 4+ | - | - |
| P9.22-47 | 37 | positive | positive | Full field of view | - | - | 1+ | - | 4+ | - | - |
| P9.25-11 | 26 | positive | positive | 1/2 field of view | - | - | 1+ | - | 3+ | - | - |
| P9.26-27 | 26 | positive | positive | a small amount | - | - | - | - | 1+ | - | - |
| P9.26-35 | 53 | positive | positive | a small amount | - | - | 1+ | - | 3+ | - |  |
| P9.26-36 | 42 | positive | positive | 1/2 field of view | - | - | 1+ | - | 3+ | - | - |
| P9.26-50 | 28 | positive | positive | 1/2 field of view | - | - | 1+ | - | 4+ | - | - |
| P9.26-69 | 40 | positive | positive | a small amount | - | - | 1+ | - | 3+ | - | - |
| P9.26-3 | 49 | positive | positive | 1/2 field of view | - | - | 1+ | - | 3+ | - | - |
| P10.7-3 | 48 | positive | positive | Full field of view | find | - | 2+ | - | 4+ | - | - |
| P10.7-38 | 29 | positive | positive | Full field of view | - | - | 1+ | - | 4+ | - | - |
| P10.7-66 | 49 | positive | positive | Full field of view | - | - | 1+ | - | 4+ | - | - |
| P10.8-47 | 44 | positive | positive | 1/2 field of view | - | - | 1+ | - | 4+ | - | - |
| P10.8-51 | 51 | positive | positive | a small amount | - | - | 2+ | - | 3+ | - | - |
| P10.8-53 | 24 | positive | positive | Full field of view | find | - | 1+ | - | 4+ | - | - |
| P10.8-69 | 30 | positive | positive | 1/2 field of view | - | - | 1+ | - | 4+ | - | - |
| P10.9-25 | 35 | positive | positive | 1/2 field of view | - | - | 1+ | - | 3+ | - | - |
| P10.9-36 | 42 | positive | positive | 1/2 field of view | - | - | 1+ | - | 4+ | - | - |
| P10.9-41 | 37 | positive | positive | Full field of view | find | - | 1+ | - | 4+ | - | - |
| P10.9-58 | 30 | positive | positive | 1/2 field of view | - | - | 1+ | - | 4+ | - | - |
| P10.9-65 | 48 | positive | positive | a small amount | - | - | 1+ | - | 2+ | - | - |
| P10.9-71 | 41 | positive | positive | 1/2 field of view | find | - | 1+ | - | 4+ | - | - |
| P10.10-28 | 52 | positive | positive | 1/2 field of view | - | - | 1+ | - | 4+ | - | - |
| P10.10-32 | 22 | positive | positive | 1/2 field of view | - | - | 3+ | - | 2+ | - | - |
| P10.10-36 | 38 | positive | positive | Full field of view | find | - | 1+ | - | 4+ | - | - |
| P10.10-54 | 27 | positive | positive | Full field of view | find | - | 1+ | - | 4+ | - | - |
| P10.11-6 | 32 | positive | positive | Full field of view | find | - | 1+ | - | 4+ | - | - |

**Supplementary Table S4** Baseline information of the population for validation.

| **No.** | **Age** | **BV Results** | **BVBlue** | **Clue Cell** | **White Blood Cell** | **Epithelial**  **Cells** | **Positive B*acilli*** | **Positive C*occi*** | **Gram- Positive *Bacillus subtilis*** | ***Corynebacterium*** |
| --- | --- | --- | --- | --- | --- | --- | --- | --- | --- | --- |
| 50_0227 | 55 | negative | negative | 0 | 15-30 | 1/2 field of view | - | ± | 4+ | - |
| 603_0227 | 49 | negative | negative | 0 | 0-5 | Full field of view | - | 0 | 4+ | - |
| 604_0227 | 44 | negative | negative | 0 | >30 | a small amount | - | 0 | 2+ | - |
| 605_0227 | 59 | negative | negative | 0 | 5-15 | 1/2 field of view | ± | 0 | - | - |
| 606_0227 | 43 | negative | negative | 0 | 15-30 | Full field of view | 4+ | 0 | - | - |
| 607_0227 | 36 | negative | negative | 0 | >30 | Full field of view | - | ± | 4+ | - |
| 609_0227 | 48 | negative | negative | 0 | >30 | 1/2 field of view | - | 1+ | 4+ | - |
| 610_0227 | 44 | negative | negative | 0 | 0-5 | 1/2 field of view | 1+ | 0 | 2+ | - |
| 611_0227 | 60 | negative | negative | 0 | >30 | Full field of view | 4+ | 0 | 0 | - |
| 51_0227 | 38 | negative | negative | 0 | 15-30 | 1/2 field of view | - | 0 | 3+ | - |
| 608_0227 | 47 | positive | positive | 0 | 0-5 | Full field of view | - | 1+ | 4+ | - |
| 27_0228 | 40 | positive | positive | 0 | 5-15 | Full field of view | - | 1+ | 2+ | 2+ |
| 33_0228 | 53 | positive | positive | 0 | >30 | 1/2 field of view | - | 1+ | 4+ | - |
| 34_0228 | 52 | positive | positive | 0 | >30 | 1/2 field of view | - | ± | 4+ | - |
| 36_0228 | 20 | positive | positive | 0 | >30 | 1/2 field of view | - | 1+ | 3+ | - |
| 37_0228 | 23 | positive | positive | 0 | >30 | 1/2 field of view | 1+ | 1+ | 3+ | - |
| 7_0229 | 32 | positive | positive | 0 | >30 | 1/2 field of view | - | ± | 4+ | - |
| 15_0229 | 49 | positive | positive | find | >30 | Full field of view | - | 1+ | 4+ | - |
| 22_0229 | 20 | positive | positive | find | 0-5 | 1/2 field of view | - | ± | 4+ | - |
| 39_0229 | 33 | positive | positive | 0 | 15-30 | 1/2 field of view | - | 0 | 4+ | - |
| 41_0305 | 41 | positive | positive | 0 | 15-30 | 1/2 field of view | - | ± | 3+ | - |
| 31_0305 | 40 | negative | negative | 0 | 0-5 | a small amount | ± | ± | - | - |
| 32_0305 | 40 | negative | negative | 0 | 5-15 | a small amount | - | ± | 2+ | - |
| 33_0305 | 33 | negative | negative | 0 | 5-15 | 1/2 field of view | 2+ | 0 | - | - |
| 34_0305 | 45 | negative | negative | 0 | 0-5 | a small amount | ± | ± | - | - |
| 40_0305 | 47 | negative | negative | 0 | 0-5 | a small amount | - | 1+ | 1+ | - |
| 42_0305 | 29 | negative | negative | 0 | 5-15 | 1/2 field of view | ± | ± | - | - |
| 43_0305 | 32 | negative | negative | 0 | 15-30 | 1/2 field of view | 2+ | 0 | - | - |
| 62_0306 | 34 | negative | negative | 0 | 5-15 | a small amount | 2+ | 0 | - | - |
| 64_0306 | 27 | negative | negative | 0 | 0-5 | a small amount | 1+ | 0 | - | - |
| 65_0306 | 36 | negative | negative | 0 | 5-15 | a small amount | 3+ | 0 | - | - |
| 76_0305 | 21 | positive | positive | 0 | >30 | 1/2 field of view | - | 1+ | 3+ | - |
| 19_0306 | 32 | positive | positive | 0 | 15-30 | 1/2 field of view | - | 1+ | 3+ | - |
| 613_0306 | 41 | positive | positive | find | 0-5 | 1/2 field of view | - | 1+ | 3+ | - |
| 63_0306 | 47 | positive | positive | find | 15-30 | 1/2 field of view | - | 1+ | 3+ | - |
| 52_0307 | 32 | positive | positive | find | >30 | 1/2 field of view | - | 1+ | 3+ | - |
| 609_0307 | 49 | positive | positive | 0 | 0-5 | a small amount | - | 1+ | 1+ | - |
| 615_0307 | 36 | positive | positive | find | 0-5 | 1/2 field of view | - | 1+ | 3+ | - |
| 618_0307 | 32 | positive | positive | find | 0-5 | 1/2 field of view | - | 1+ | 3+ | - |
| 628_0307 | 47 | positive | positive | 0 | 15-30 | 1/2 field of view | - | 1+ | 3+ | - |
| 607_0308 | 27 | positive | positive | find | 15-30 | 1/2 field of view | - | 1+ | 2+ | - |
